# Leveraging machine learning and prescriptive analytics to improve operating room throughput

**DOI:** 10.3389/fdgth.2023.1242214

**Published:** 2023-09-22

**Authors:** Farid Al Zoubi, Georges Khalaf, Paul E. Beaulé, Pascal Fallavollita

**Affiliations:** ^1^ School of Electrical Engineering and Computer Science, University of Ottawa, Ottawa, ON, Canada; ^2^ The Ottawa-Carleton Institute of Biomedical Engineering (OCIBME), University of Ottawa, Ottawa, ON, Canada; ^3^ Division of Orthopedic Surgery, Ottawa Hospital Research Institute, Ottawa, ON, Canada; ^4^ Interdisciplinary School of Health Sciences, University of Ottawa, Ottawa, ON, Canada

**Keywords:** prescriptive analytics, predictive analytics, machine learning, time forecast, health care efficiency, high volume surgery, operating room throughput

## Abstract

Successful days are defined as days when four cases were completed before 3:45pm, and overtime hours are defined as time spent after 3:45pm. Based on these definitions and the 460 unsuccessful days isolated from the dataset, 465 hours, 22 minutes, and 30 seconds total overtime hours were calculated. To reduce the increasing wait lists for hip and knee surgeries, we aim to verify whether it is possible to add a 5th surgery, to the typical 4 arthroplasty surgery per day schedule, without adding extra overtime hours and cost at our clinical institution. To predict 5th cases, 301 successful days were isolated and used to fit linear regression models for each individual day. After using the models' predictions, it was determined that increasing performance to a 77% success rate can lead to approximately 35 extra cases per year, while performing optimally at a 100% success rate can translate to 56 extra cases per year at no extra cost. Overall, this shows the extent of resources wasted by overtime costs, and the potential for their use in reducing long wait times. Future work can explore optimal staffing procedures to account for these extra cases.

## 1. Introduction

In Canada, the median wait time for treatment from referral by a general practitioner (GP) was 27.4 weeks (12.6 from GP to specialist, and 14.8 from specialist to treatment) in 2022. This value continues to trend upwards, even relative to pre-determined reasonable wait times (1).When looking at individual specialties, orthopedic surgery not only consistently demonstrates long median wait times over several years, but also has the longest median wait time from specialist to treatment in 2021 (30.2 weeks) and is second only to plastic surgery in 2022 (32.4 vs. 34.3 weeks) (1). Despite having an estimated median reasonable wait time of 15.4 weeks, hip and knee replacement surgeries were still given a Pan-Canadian benchmark wait time of 26 weeks as a maximum, yet it is still lower than the national median wait time of 38.0 weeks in 2022 (1). In Ontario alone, there are currently an estimated 206,000 patients waiting for surgical procedures (2). For orthopedic surgery in Ontario, the median waiting time is 19.9 weeks, 75% greater than the province's reasonable median wait time of 11.4 weeks, leaving an estimated 38,275 patients waiting for orthopedic treatment, 25,372 of which are for arthroplasty surgeries (1).

Before the COVID-19 pandemic, hip and knee replacements were increasing at a rate of 5% per year. During the COVID-19 pandemic, hip and knee replacements between April and December decreased by 16.1% and 29.8% respectively from 2019 to 2020 due to an abundance of cancellations, creating an excess of waiting patients. In fact, current trends in Canada have led to 138,500 surgeries and estimated inpatient costs of over $1.4 billion a year, imposing a huge burden on the economy, in addition to large backlogs in waiting lists (3). Unfortunately, data indicates that in order to overcome these large backlogs, provinces will need to exceed pre-pandemic rates of surgery, something that has only been accomplished 3 times nationally since the beginning of the pandemic (4).

In Ontario, patients are triaged into different categories based on urgency: priority 4 patients have a target treatment time of 182 days, priority 3 have a target treatment time of 84 days, and priority 2 patients should be treated within 42 days. When evaluating the wait time from the decision for surgery to the surgery itself, only 16% of hip replacement patients and 10% of knee replacement patients are treated within the target time at our institution. For the former treatment, the average wait time for priority 4 patients is 375 days, while that for priority 2 patients is 135 days (not enough data for priority 3), while knee replacement patients yield average wait times of 398 days for priority 4 patients, and 214 days for priority 3 patients (not enough data for priority 2) (5).

Current options that support and enhance the scheduling and efficiency of hospitals come in two main types: clinical and non-clinical methods. The non-clinical methods (6, 7) can be divided into three approaches: firstly, incorporating new resources to enhance the effectiveness of operating rooms (8–11); secondly, utilizing data-driven methods like rhetorical data and descriptive analytics to modify, assess, or reorganize existing hospital resources (12, 13); and thirdly, employing machine learning (ML) solutions specifically designed for operating room optimization. The majority of these solutions have the common goal of foreseeing various aspects of surgical procedures. This involves predicting events both before (14) and after surgery (14–16), as well as accurately estimating the duration of the surgical process itself (17–19).

Expenses can be associated with any of these phases of surgery, which opens up opportunities for cost reduction (20). One way to achieve this is by diminishing the necessary resources through the implementation of self-management application (21). Additionally, certain decision support systems have been employed to simulate various scenarios involving interactions with staff and managers, potentially leading to an increase in the number of cases on certain days (22).

Other Efforts such as 4-joint operating rooms (OR) (i.e., dedicated to serving 4 operations within 8 h) have been implemented to increase throughput and shrink existing waiting lists (23). However, the issue persisted, as the 4-joint room was only able to report a 49% success rate in 2012, indicating a lack of consistent efficiency (23). This is additionally concerning as inefficient use of resources and time contribute to 30% of total healthcare expenditures (14), further emphasizing the need to optimize time and cost. These numbers highlight the burden placed on this hospital, and the pressing need for solutions to reduce the waiting list.

We are of the opinion that we are pioneering the utilization of perspective analytics to compute the expenses linked to overtime pay and forecast whether this sum suffices to accommodate a fifth instance of a 4-joint arthroplasty procedure.

## 2. Previous work

**Figure 1:**
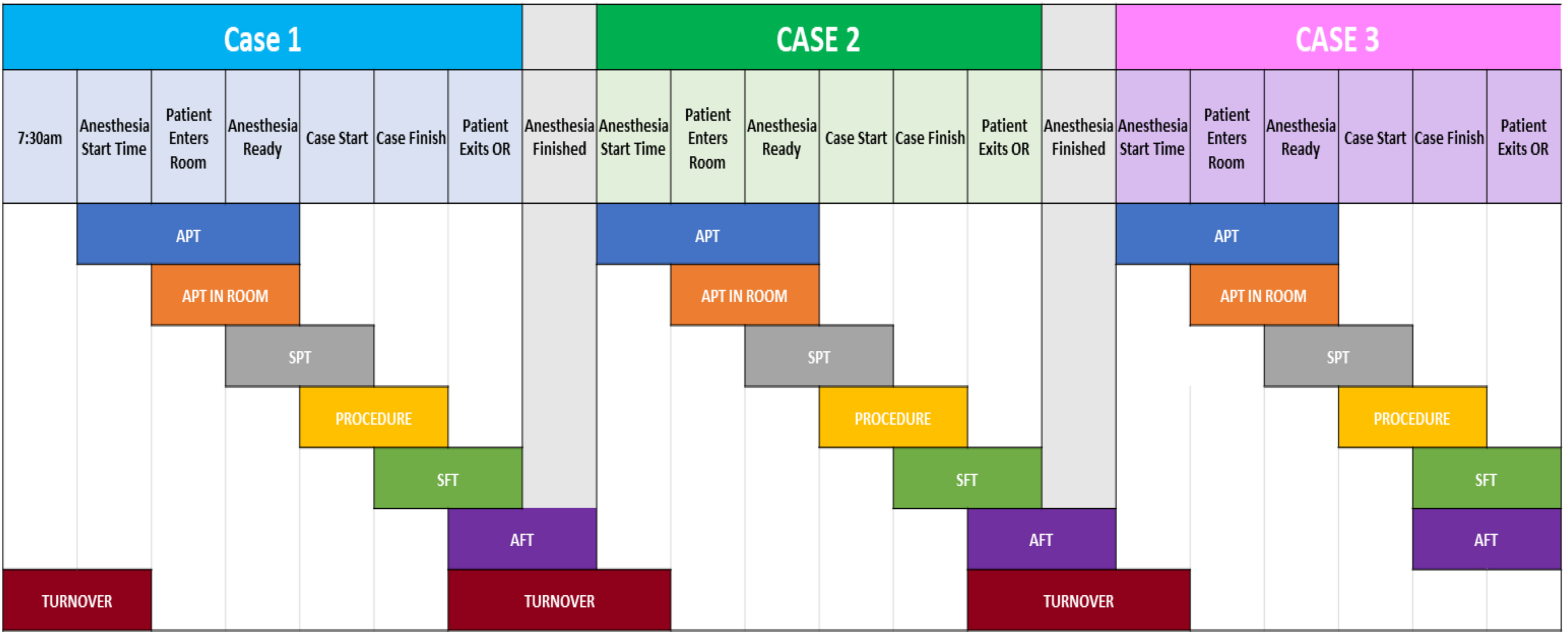
An overview of each stage of a procedure, as well as the transition between two (turnover). At the top are the stage markers of a case, while the black region shows the different duration variables used. Acronyms are fully explained in Table 3.

Our institution has a dedicated orthopedic OR for 4-joint arthroplasty procedures. The OR is specially designed for high-volume arthroplasty surgeries, i.e., partial, and complete joint replacement, and facilitates four procedures each day (from Monday to Friday, excluding Wednesday at which time the ORs start 30 min later for education). Each procedure is subdivided into six stages, including Anesthesia Preparation, patient positioning, surgical procedure, patient exiting the room and turnover, the final stage (see Figure 1).

A successful day in this arthroplasty OR is defined as the completion of all four procedures within the allocated time; in this case, the eight hours assigned between 7:30 am and 3:30 pm; however, because there is a 15-min buffer window for overtime pay at our institution, 3:45 pm is used in the proposed methods of this article. The Surgical Success Rate or SSR was the metric designed to keep track of the percentage of successful days in a predetermined period (typically a year).

The original SSR was dismal - 39%, and the overtime cost for our institution was roughly $570,000 annually. Multiple initiatives were introduced to improve this SSR with varying degrees of success (23–25).

**Figure 2:**
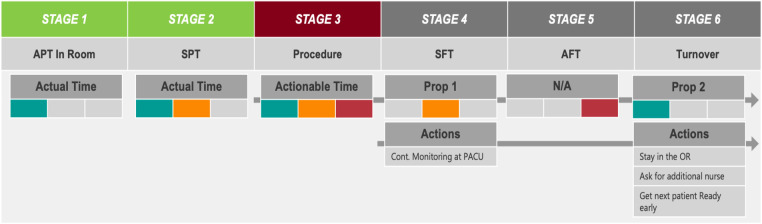
A visual demonstration of the artificial intelligence model's function. Colors are used to indicate timeliness at each stage of the procedure, while suggestions are provided for ways to maintain or catch up to the desired rate.

Recently, we suggested the most comprehensive solution to this problem—a data-driven, Machine Learning (ML)-based, prescriptive analytics system. It not only predicts the probability of whether a particular day would be successful based on time variables, but also monitors each stage of the procedure in real-time, modifying its prediction if needed, and offers suggestions through a proposed list of actions at a given stage to increase the probability of success (25), as demonstrated in Figure 2.

These suggested actions are updated stage-by-stage for each procedure. The multiplicity of suggestions ensures that the surgical team has multiple viable options to choose from, allowing them to leverage their experience and expertise to employ what they believe would be the most positively impactful suggestion in any given circumstance (see Figure 2). These real-time proposals allow the system to not just monitor but optimize the procedures and influence the SSR.

The suggestions offered by the ML-based prescriptive analytics system were developed and tested during a comprehensive and highly successful program designed to optimize OR procedures. The program focused on Positive Deviance seminars offered by the most successful surgical professionals in their respective domains. This included surgeons and registered nurses (RNs) with the highest SSR, with the idea of sharing their expertise and best practices with the team to improve overall SSR (8). At our center, during the PD exercise the anesthesiologists refused to participate.

These professionals shared the processes and procedure optimization techniques that allowed them to complete all four surgeries on time (without compromising patient safety) with the rest of the team (see Table 1). These processes and techniques were made part of the ML system, which would suggest the right set of actions at various stages of the procedure to optimize the process.

**Table 1:** Examples of suggestions offered by surgeons and nurses at positive deviance seminars to optimize OR procedures.

Surgeons	Nursing
1. Be there from positioning to patient transfer from the table. 2. Have a standardized/protocolized approach for each type of procedure. 3. Anticipate next steps, calling for instruments/implants. 4. Assist with turnover and putting away instruments, but in a way that is supported by nurses. 5. Institute an incentivization for the entire team to be done by 3:30, and that would drive efficiency. 6. Bring the patient into the room for spinal preparation such that instruments may be opened simultaneously (in parallel rather than in series) 7. Anesthesia does the blocks and spinals in the procedure room.	1. Have an engaged, familiar team working together. 2. Have equipment ready to go before patient enters the room. 3. Whole team (nursing, surgery, anesthesia) is present during turnover. 4. Begin putting away instrumentation during closing. 5. Have experienced, knowledgeable scrub nurses who know the steps to the procedure and will know when certain instruments (implants) are needed. 6. Have attendants available to help with turnover. 7. Ensure nurses in the room have received total joint training. 8. Minimize phone call interruptions from pre-op and PACU during the case. 9. Ensure attending available for prep. Make use of free staff in room when prepping/positioning. Ensure no revision of surgical positioning. 10. Need team lead (TL) to have adequate time for training and administration. 11. Ensure improvements in efficiency don’t come at cost to patient outcomes.

Following the system's suggestions resulted in a significant improvement in individual procedure times and, as a result, an improvement in the overall SSR.

### 2.1. The benchmarks

The model establishes multiple sets of benchmarks to track success and failure by monitoring the six stages of individual procedure case. More specifically, both stage duration benchmarks and recommendations are produced for any desired SSR. Based on the sets produced, a 77% success rate was defined as the baseline, replacing the default SSR of 39% (25). This SSR was selected as the baseline because its benchmarks were deemed easily achievable by the clinicians and through the leveraging of the prescriptive analytics system (see Table 2). In doing so, it leads to improve nearly three out of five (61%) failed days on which the 4 surgeries were not completed on time. These failed days, on average, cost the hospital about 36 min of overtime (more than $2,000 a day).

**Table 2:** Benchmarks established by the AI model for different success rates. The baseline and the optimal scenarios are used for evaluation in this paper.

Scenario	APT (mins)	Case (mins)	AFT (mins)	Turnover (mins)	Success rate
Baseline (75th percentile)	<10.5	<71.5	<20.5	<21.5	77%
Fast procedure	<10.5	53	<20.5	<21.5	93%
	<10.5	64	<20.5	<21.5	89%
Slow procedure	<10.5	>71.5	<20.5	<21.5	59%
Slow turnover	<10.5	<71.5	<20.5	>21.5	69%
Slow anesthesia Preparation	10.5–18.5	<71.6	<20.5	<21.5	64%
Optimal performance	<7	<62.5	≤7	≤20	100%

Another benchmark is the best-case scenario - 100%. This is achievable when the prescriptive analytics system is fully leveraged, and the most potent actions/suggestions are followed to optimize the overall procedure time. This results not only in a successful day, but also with an adequate amount of time left within the eight-hour window. It is this scenario that encouraged us to suggest a follow-up model. Example benchmarks for this scenario as well as others are shown in Table 2.

## 3. Relation to this work

With the development of our ML-based prescriptive analytics system, the ideal scenario would be zero overtime and all surgeries completed on time on any given day. However, even if we disregard the anomalies during procedure and preparation, there are several factors preventing this ideal scenario from becoming the norm, including a limited number of high performers and the inevitable concentration of more time-consuming patients on certain days (statistically significant).

**Figure 3:**
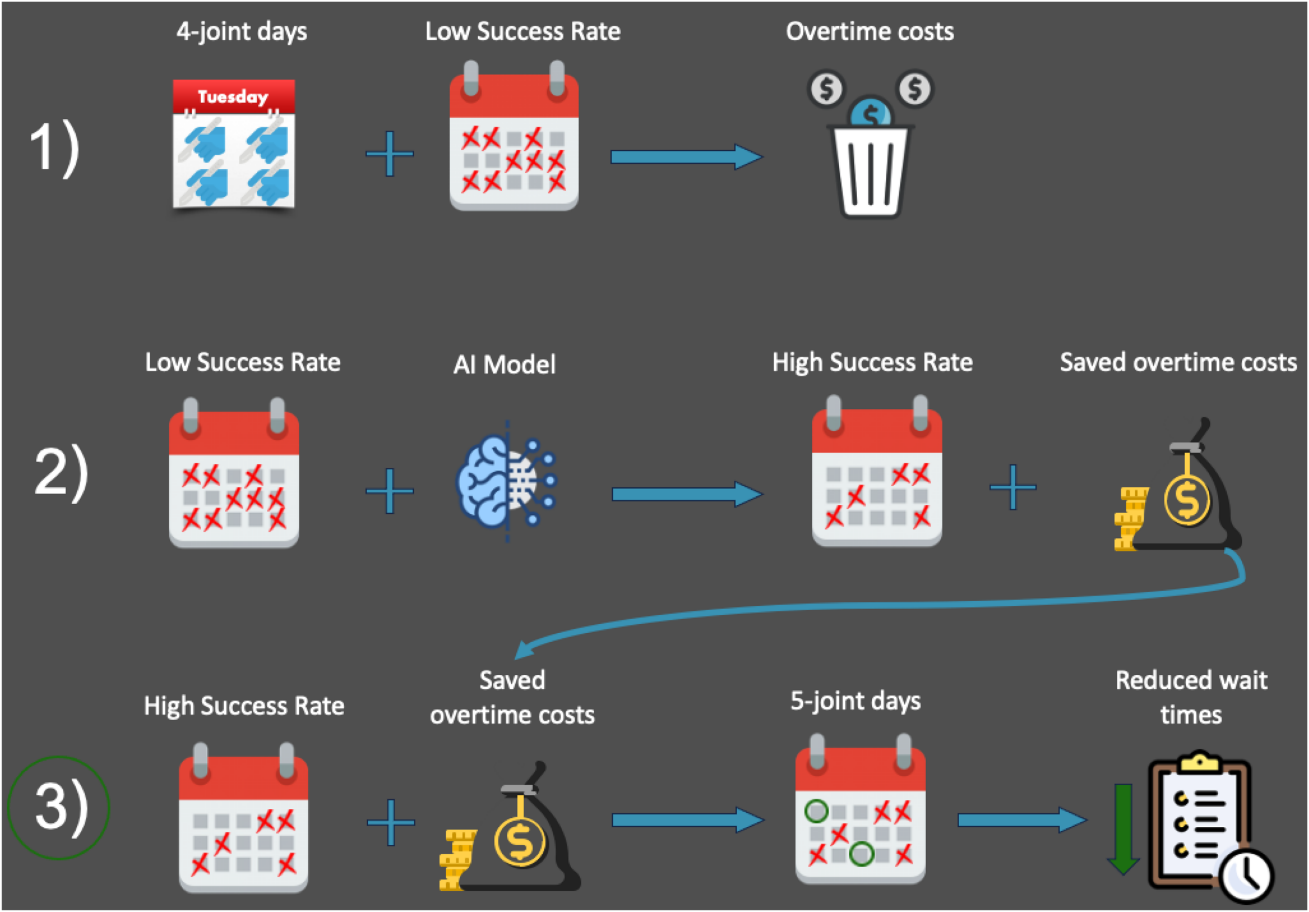
A flowchart showing an overview of our research plan. This article focuses on row 3.

This encouraged us to consider the above two benchmarks, 77% and 100% SSR outputs of our perspective system to predict the possibility of completing an additional surgery (i.e., a 5th case) during a successful day. To do this, savings from the decrease in overtime hours and its increased pay will be evaluated for its ability to fund the 5th cases, leading to an increase in throughput with no extra cost. This will ensure fair compensation to the surgical staff for a higher number of overall surgeries because additional surgeries would be covered under the saved overtime that the staff has already been paid for. A flowchart visualizing this process can be found in Figure 3.

Even with a rudimentary calculation, the average overtime for four unsuccessful days (36 min times four) will be roughly enough to justify fitting in a fifth surgery on a successful day. With the cost and time justified, the analytics systems will be applied to different time distribution scenarios to identify how many successful five-surgery days are feasible and justifiable. This may require us to consolidate recommendations like scheduling multiple low-risk/less-time-consuming patients on a single day or deferring monitoring in the Post-Anesthesia Care Unit (PACU).

Therefore, the contribution of this article is to implement a predictive method to estimate of likelihood of fitting a 5th=surgery during a successful 4-joint operating room day, using only cost-savings accrued from use of our previous work—an Artificial Intelligence (AI)-based model that produces time-based benchmarks for different success rates. Cost savings from two success rates that will be evaluated: 77%, our realistic baseline, and 100%, optimal performance (25). To do this, we will utilize linear regression by fitting a model to every successful day, generating a histogram of 5th case predictions that will be leveraged to explore different distribution strategies of saved costs.

## 4. Arthroplasty data set

In order to leverage the existing ML-based prescriptive analytics system for the proposed fifth case simulation, it's imperative to understand the foundational data used to build the original model/system.

### 4.1. Time period

The data collected for building use in this paper spans from 2012 to 2019. This is enough time to recognize almost all short-term and long-term patterns in data. The statistically significant amount of data naturally lowered variance estimation, leading to more accurate predictions and, consequently, drawing more relevant recommendations.

### 4.2. Nature of procedures

To streamline the data and identify better optimization techniques, we adhered to non-complex cases and unilateral surgeries. Our early analyses found that bilateral surgeries take more time, and even if it is not twice as long as unilateral surgeries (such as the actual difference in the surgical procedure), it is statistically significant enough to prevent the on-time completion of four surgeries in a day. It is also important to consider that typically, there is an approximate 4:1 unilateral to bilateral surgery ratio, so the bulk of the procedures were considered. Similarly, complex surgeries where health complications, which may prolong the procedure, are identified beforehand were also excluded from the data set since they are predictable rarities, not the norm.

### 4.3. Nature of data

The data our machine-learning systems were trained on was both numerical and categorical in nature. The numerical data mostly consisted of timestamps for every stage of the procedure, which were converted to durations to generate a rich number of numerical variables and metrics. The categorical data came from the individuals and the type of surgeries performed. Collectively, the data pool consisting of 40 different variables covering almost all medically relevant details about the patient and procedure, the surgical team performing the procedure, and the necessary time variables; however, the final dataset was filtered down, resulting in 29 of the pool's 40 variables being used (see Table 3). Of these 29, Case Number and Out of Room Time will be used in this paper's models, highlighted in Table 3.

**Table 3:** The selected variables that were present in the dataset used for the development of the AI model.

Time metrics	Staff (team) metrics	Patient metrics	Safety metrics
Anesthesia preparation time (APT)	Surgeon	Campus	90-day readmissions
Anesthesia start time	Anesthesiologist	Type of surgery	Reason for readmission
Time in room	Circulator Nurse 1	Type of anesthesia	Length of stay
Anesthesia ready time	Circulator Nurse 2	Sex	
Anesthesia stop time		Age	
Anesthesia finish time (AFT)		BMI	
Surgical preparation time (SPT)		ASA	
Case start			
Case finish			
Surgery finish time (SFT)			
Turnover			
Surgery (procedure) time			
Time out of room			
Case no			
Date			

Time out of Room and Case No are the features used for this work.

### 4.4. Data collection source

The data for most of the identified metrics came from the Surgical Information Management System (SIMS), though some data points came from patient charts and daily notes. This consolidated sourcing of the relevant data prevented the need for integrating different information management systems and overcomplicating the process. This is also one of the factors making this system extrapolatable to different healthcare facilities.

### 4.5. Treatment of data

Since we already removed anomalies like complicated cases and statistical outliers (bilateral surgeries) that would undermine the pattern recognition and generation of useful insights, the treatment was relatively minimal. The data was cleaned for missing information and incorrect values, both of which represented less than 1% of the observed data set, so the removal was not significant enough to impact the statistical outcome. Regardless of their dissent with median values, rare cases (categorized as non-complex before the procedure began) were kept in the data set. They had a modest impact on the extremes, but not enough to deviate from the trends enough to draw wrong conclusions.

### 4.6. Demographics of patients and other quantifiable

Table 4 contains required information about demographics of patients and some other quantifiable.

**Table 4:** General patient demographics from the sample dataset.

Number of surgery days (total surgeries)	761 (3,044)
Distribution of male and female patients	1,560 (51.25%) M; 1,484 (48.75%) F
Average patient age	63.2 ± 11.9
Average patient BMI	30 ± 5

### 4.7. Descriptive analytics

Of the 761 4-joint operation days, 301 were successful (4 operations before 15:45), marking a 39.55% success rate. In these successful days, there was a total 97 h and 49 min of spare time (time between the end of the final case and 15:45), averaging to 19 min and 30 s per day. Overtime-cost hours were calculated using the remaining 460 unsuccessful days by multiplying the number of overtime hours (hours worked past 15:45) by 1.5 (the paid overtime rate). Doing so reveals a total 465 h, 22 min, and 30 s overtime-cost hours, leading to an average of one hour per unsuccessful day.

**Figure 4:**
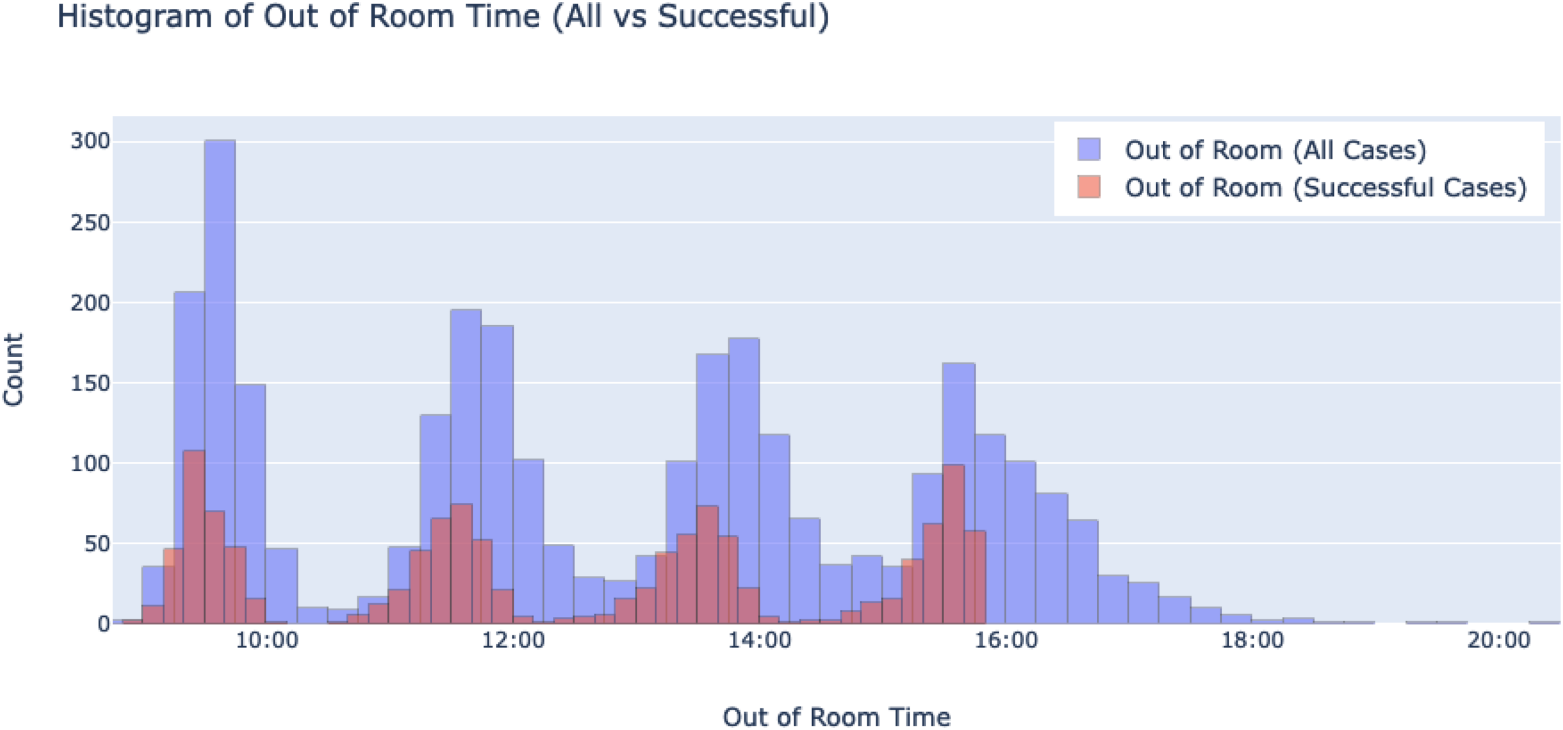
A histogram of out of room times showing all individual cases, and successful cases. The x-axis shows bins of times at which patients have been escorted out of the operation room, while the y-axis shows the number of cases that are in each bin.

Each case's out of room time, which is the time at which the patient is taken out of the operating room, is plotted on a histogram, showing distributions of successful cases and all cases (see Figure 4). Peaks on the graph belong to individual distributions of one of the four cases in a day. The distribution of all 4th cases shows a tail that extends past 8 pm, marking over 4 h of overtime on some days. In general, the skew of all cases appears to increase to the right with each subsequent case number, while that of successful cases appear to do so minimally to the left. In line with the trend of increasing skews, the spread of each distribution also increases with each subsequent case number, going from having a standard deviation of 00:17 m:44 s for first cases to 00:42 m:05 s for fourth cases, as shown in Table 5. Table 5 also shows a stark difference between successful cases and all cases, as the former is much more consistent with their times, as demonstrated by their smaller standard deviations.

**Table 5:** The standard deviations of successful cases and all cases.

	All cases	Successful cases
1st case	00:17:44	00:12:07
2nd case	00:26:14	00:17:01
3rd case	00:31:26	00:18:56
4th case	00:42:05	00:16:51*

* Reduced by a cut of the distribution due to the limit at 15:45.

**Figure 5:**
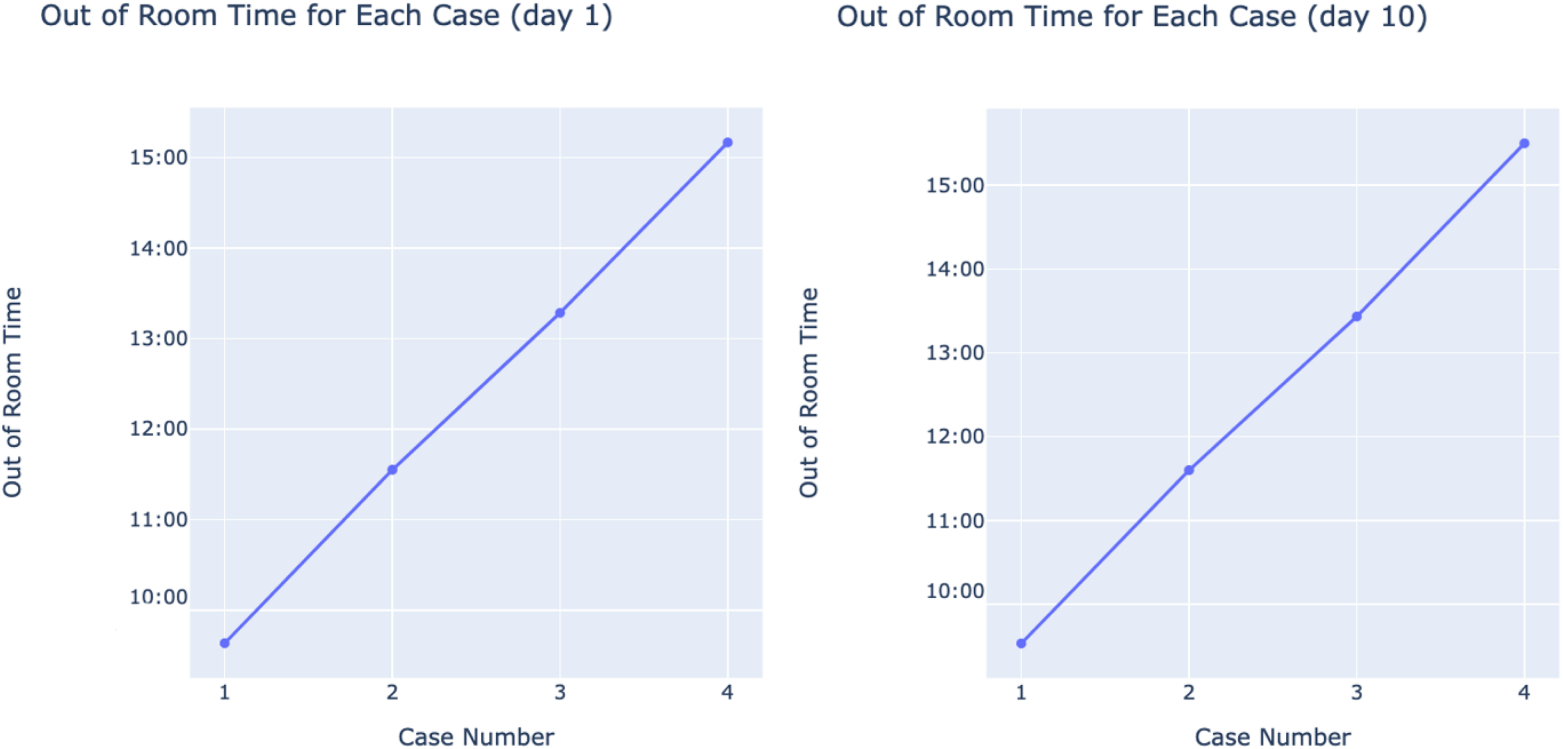
Random examples of successful days’ out of room times with case numbers on *x* and out of room times on *y*.

Individual successful days were isolated, and their out of room times were each graphed against their case number. Figure 5 shows an example of 4 different days, all of which demonstrate the linear nature of out of room times for a specific day. This observed trend inspired our method of predicting 5th cases as described in the Methodology.

## 5. Methodology

The data was first divided into successful and unsuccessful groups: the successful group contains all days where the fourth case was completed (as defined by its out of room time) at or before 15:45, while the unsuccessful group contained all days where the 4th case was completed after 15:45.

As previously mentioned, there is an average of 19 min and 30 s of spare time per successful day; to ensure that this spare time is used and that staff do not end their days later than needed, it was decided that 5th cases will only be added to successful days. This decision is further reinforced by the successful group's more consistent trends, which facilitates model training to yield more accurate results. Thus, the successful group is used for the prediction of 5th cases and their potential addition, while the unsuccessful group is used for the calculation of overtime-cost hours and the distribution of their hypothetical savings among all days.

**Figure 6:**
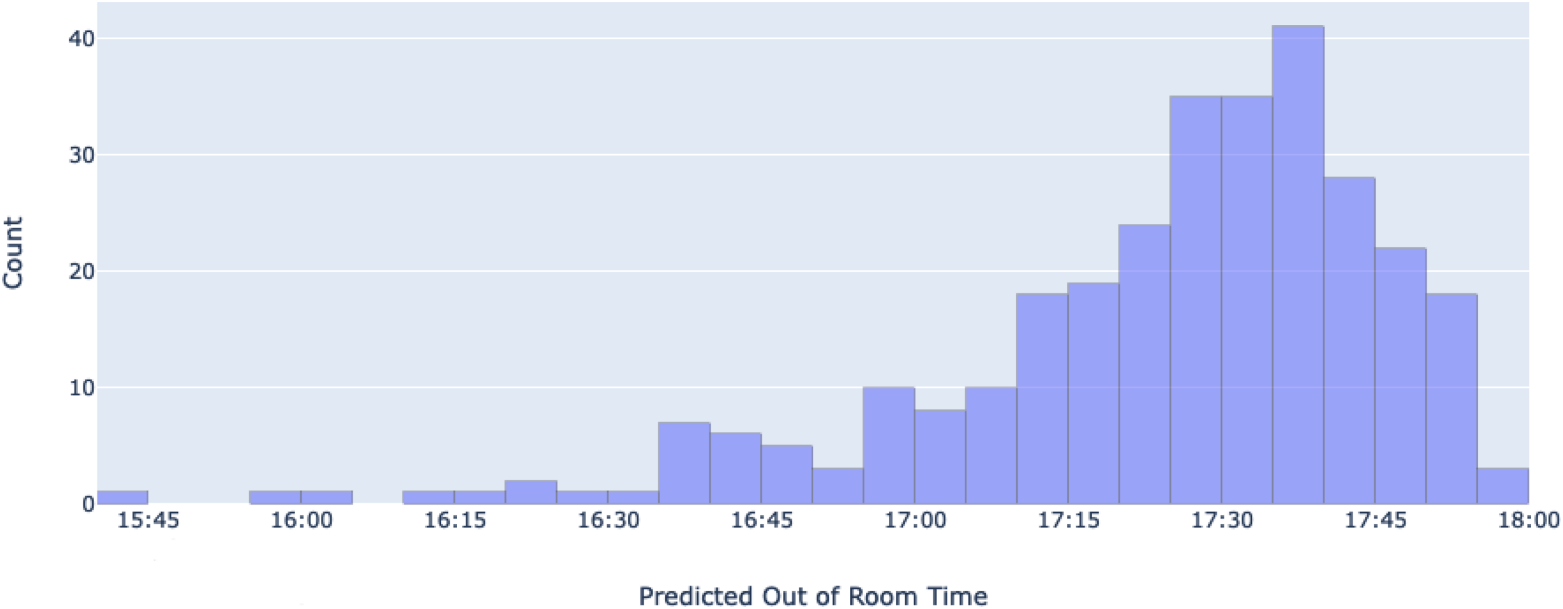
A histogram of all predicted 5th case out of room times. The x-axis represents different bins of predicted 5th case out of room times (time at which patients are escorted out of the operation room), while the y-axis shows the number of predicted cases that are in each bin.

To predict 5th case out of room times, the linear nature of each successful day's cases is leveraged (see Figure 5). All 301 successful days are isolated, along with their 4 cases. For each isolated day, a linear regression model is used to fit the out of room times with case number as the independent variable, and a 5th case prediction is generated from each one. Following the prediction for each day, a distribution of 5th case predictions is produced (shown in Figure 6). This distribution will be used to evaluate the potential of adding extra cases by using previous cost savings calculated from unsuccessful days. The use of linear regression is advantageous due to its interpretability, simplicity, and its ability to make predictions without ground truth data. Due to the nature of the problem, no 5th case ground truth data is available, limiting the scope of methods to choose from. Linear regression overcomes this problem by not requiring the desired input to be in the training set. Given the approach of isolating successful days, each day's model can be individually analyzed and adjusted, with its slope representing that day's average case duration. Further, linear regression accounts for the day's start time by accounting for the first case, and naturally produces variability in case durations without compromising overall accuracy by fitting to the existing variability within the dataset. Finally, this method allows for the prediction of further cases (ex. 6th, 7th, etc.) if needed by simply changing the input variable. The linear regression equation is shown below, where $w_{1}$ denote the trainable weights, *x* is the inputted case number, and *y* is the associated Out of Room time. Practically, $w_{1}$ reveals a given day's average case duration, while $w_{0}$ accounts for varying start times between days.


(1) $$y=w_{1}x+\;w_{0}$$

Calculations for overtime-cost hours saved (OCHS) were made using this equation:

(2) OCHS=465.375−761x(1−y)

Where *x* is the previously calculated overtime-cost hours per unsuccessful day, *y* is the new success rate, 465.375 represents the current number of overtime-cost hours (based on the 39.55% success rate), and 761 is the total number of days in the dataset. Thus, a 100% success rate would lead to saving 465.375 overtime-cost hours. The calculated OCHS is then divided in 3 ways: daily (761 days), bi-daily, and once a week (4-day work weeks), each producing a different 5th case success time benchmark. Once done, the predicted 5th cases are used to generate success rates for each success time and calculate the number of potential extra cases delivered at no extra cost.

## 6. Validation

Because there is no ground truth data, two methods of validation are employed. Both methods rely on Mean Absolute Error (MAE), which is described by the following equation:

(3) $$\mathrm{MAE}=\;\frac{1}{n}\overset{n}{\underset{i=1}{\sum }}|y_{i}-{\hat{y}}_{i}|$$

Where *n* is the sample size, *y* is the actual value, and $\hat{y}$ is the predicted value. MAE is used because of its interpretability, as it provides the actual mean time difference between the generated values and the ground truth.

The first method is the prediction of 3rd and 4th case out of room times, so that MAEs can be generated from their existing ground truth data. To do this, the same procedure that is used to predict 5th cases is also used to predict 3rd and 4th, with the difference being that the lines are only fit to the first 2 and 3 cases of each successful day respectively. Once all the errors are calculated, histograms and 95% mean confidence intervals are produced for the MAEs to gain a deeper understanding of the model's performance. Through this method, we are leveraging existing ground truth within the data to produce MAEs that can be used to infer that of the prediction of 5th cases.

The second method is used to give an impression of how well the lines fit to the existing data. Each day's model has their MAE calculated using the points on the line and the actual 4 case out of room times. From there, another distribution and 95% mean confidence interval is generated for further insight into linear regression's performance on the dataset, as well as the 5th case predictions' errors themselves.

## 7. Results

All predictions were compiled and visualized as a distribution (Figure 6). The predictions have a mean time of 17:24:17 (95% CI = 17:21:46, 17:26:48) a median of 17:29:30, and a standard deviation of 22 min and 10 s. Of the 301 predictions, 256 of them (85.7%) fall below the 2-h mark (17:45), while all 301 (100%) are predicted to end before 18:00, as the latest predicted time is 17:56:30.

**Figure 7:**
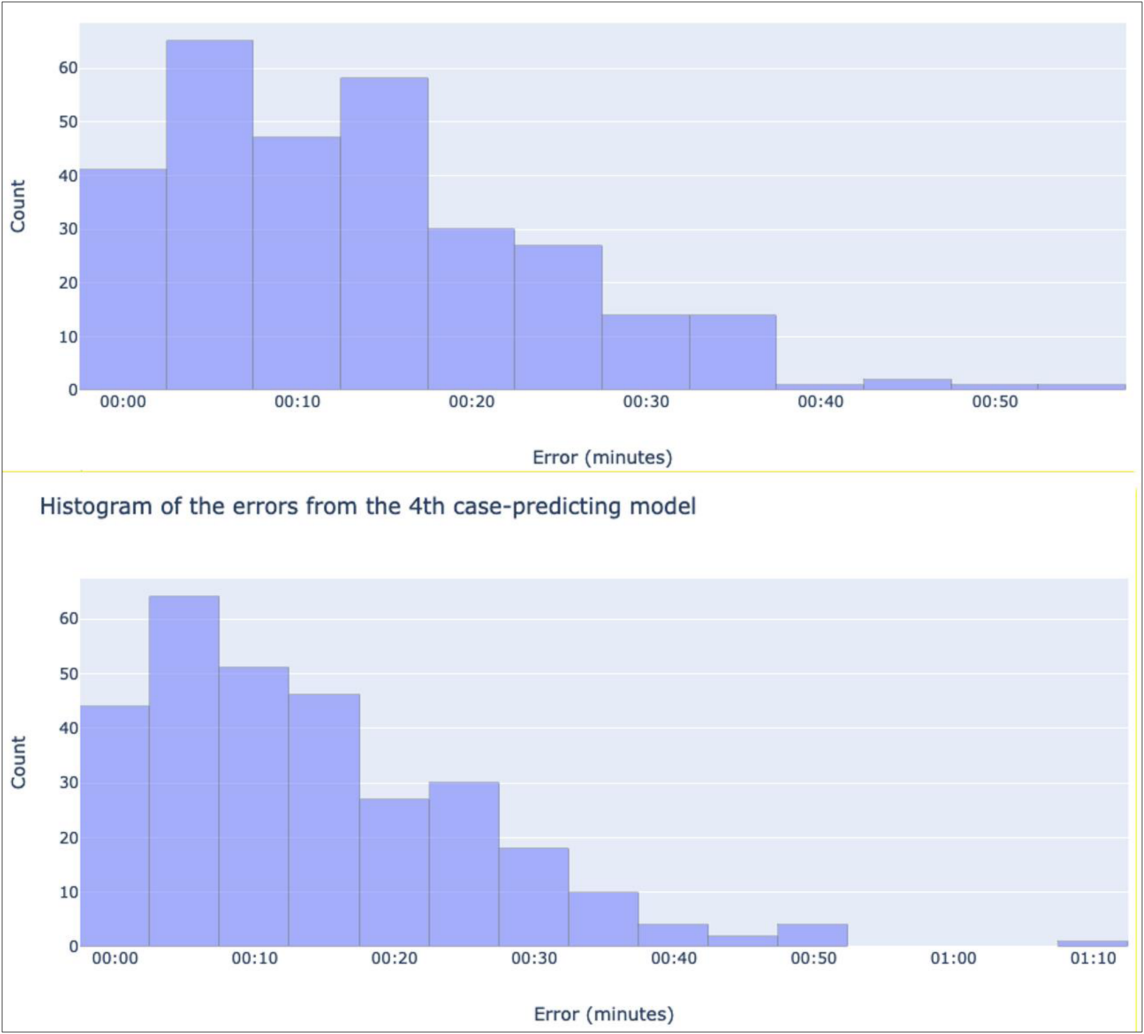
Histograms showing the distribution of the absolute values of each recorded error for the 3rd case-predicting model (top), and the 4th-case predicting model (bottom). The x-axes denote bins of absolute error values in minutes, while the y-axes show the number of predictions in each bin.

After training 3rd and 4th case-predicting models, mean absolute error values of 13 m:40 s and 14 m:13 s minutes respectively were calculated. A distribution of every day's error for each model is shown in Figure 7. These distributions yield 95% mean confidence intervals of 00 h:12 m:28 s, 00 h:14 m:50 s and 00 h:12 m:53 s, 00 h:15 m:31 s for the 3rd and 4th case models respectively.

**Figure 8:**
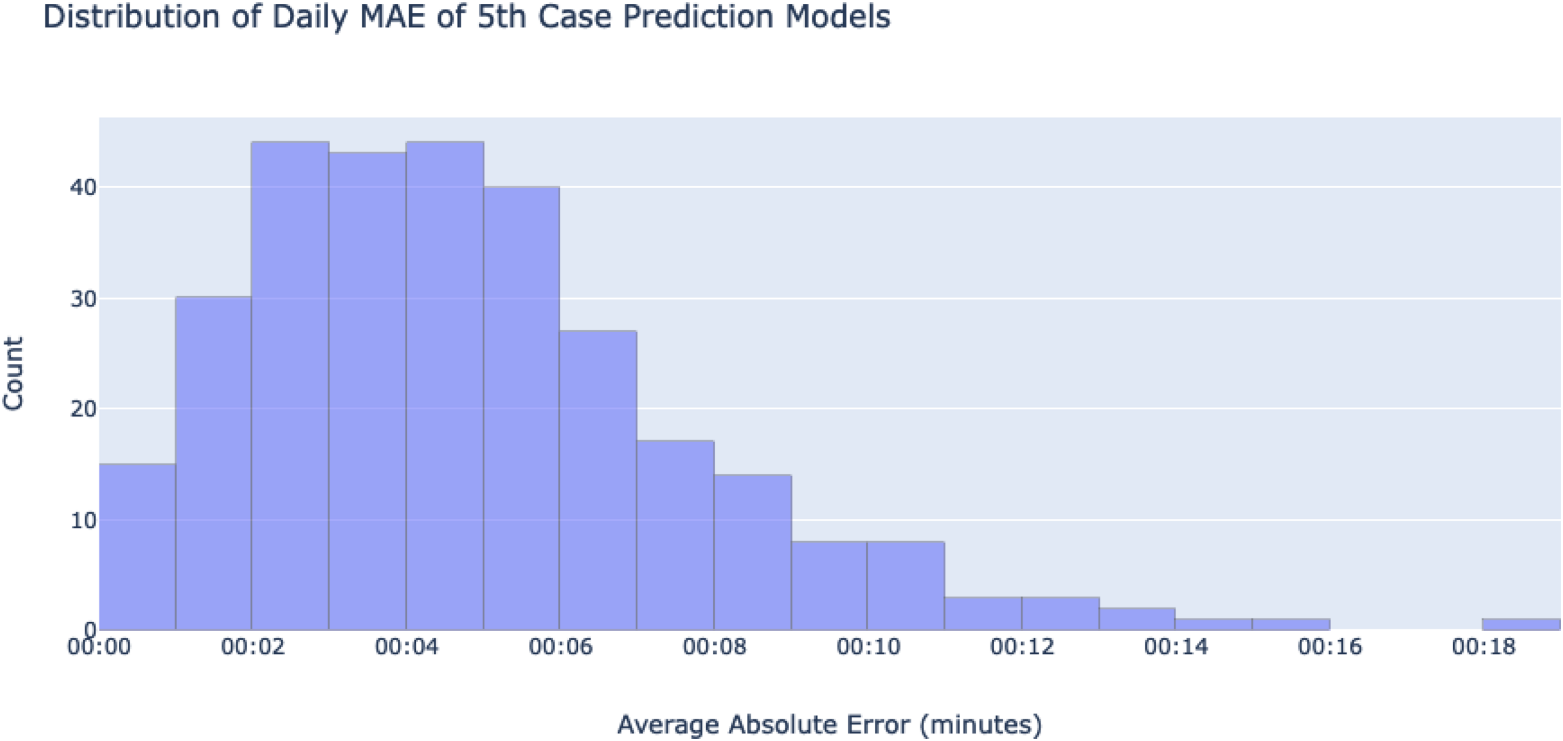
A histogram showing a distribution of the absolute values of each day's MAE. MAEs were calculated using the differences of each ground-truth case and their fitted lines. The x-axis shows bins of mean absolute errors for any given day's 5th case-predicting model, while the y-axis counts the number of days in each bin.

Similar outputs were produced for the second method; the mean of all daily MAEs was 4:45 min, with a 95% mean confidence interval of 00 h:04 m:25 s, 00 h:05 m:05 s and a standard deviation of 2:55 min. The histogram of all daily MAEs can be found at Figure 8.

## 8. Linear regression compared to other techniques

While evaluating different approaches, the characteristics of our dataset significantly limited the choices available to us. Firstly, the absence of ground truth data for the fifth case eliminated the option of employing machine learning techniques reliant on labeled training data. Secondly, we identified a pronounced linear pattern in successful outcomes, prompting the adoption of linear regression. Although we explored alternative regression methodologies, it became evident that none were viable for our particular scenario where each day required a distinct model. Bayesian regression, for instance, necessitated data distributions to derive information, but this method proved unsuitable given the small dataset of four data points per model across each day.

Given the solvability of linear regression, we disregarded models employing gradient descent or regularization techniques as unnecessary. Nonetheless, for the purpose of comparative analysis, we contemplated incorporating the average duration of each case (119 min). To achieve this, we added the time taken for the fourth case from the available room time and assigned this duration to the fifth case, assuming a success time of 5:45 pm. This hypothetical adjustment yielded a 100% success rate, as all fourth cases concluded prior to 3:45 pm. It is important to note that this outcome is unrealistic and contradicts our understanding of the situation. Furthermore, the identical distribution between the fourth and fifth cases is also unrealistic, particularly when considering that the standard deviation increases with each successive case number.

## 9. Predicting the potential to Fit a 5th case during successful surgery days

**Figure 9:**
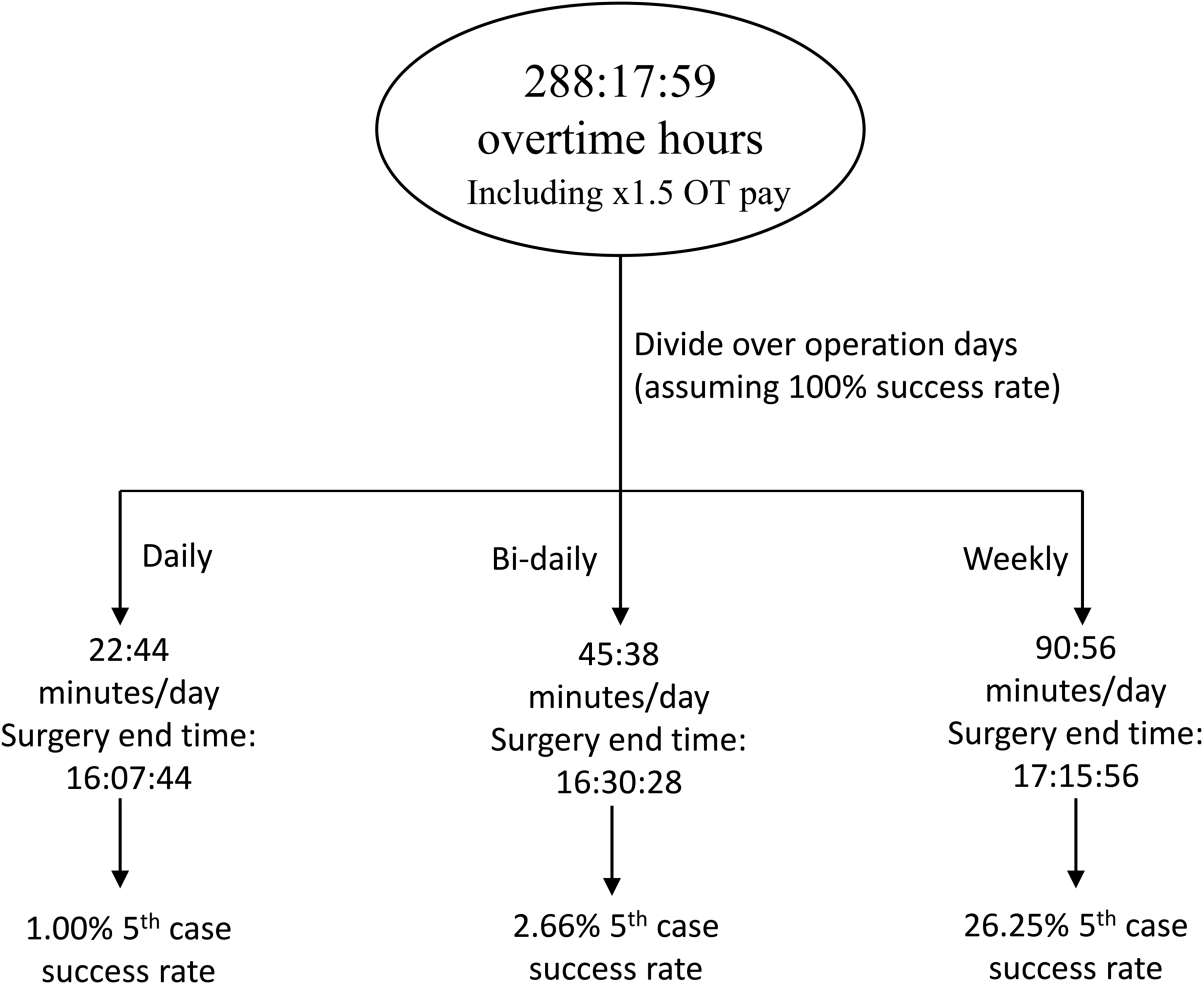
A schematic demonstrating the division of saved overtime-cost hours from performance at a 77% success rate. There is only a 1% chance of fitting a 5th case successfully each day, and up to 26.25% chance to add a 5th case per week.

The predictions were considered under two potential scenarios: 77% success rate, and 100% success rate. Using Formula 2, achieving a 77% success rate would yield hypothetical savings of 288 h:17 m:50 s overtime-cost hours, which is approximately 38 h and 26 min per year. Distributing these hours daily leads to 22 m:44 s extra minutes per 5th case day, which when added to the original end time of 15:45, would produce a new end time of 16h:07 m:44 s. Based on predictions, 5th cases would be completed at a 1.00% success rate for that time. This extra time is doubled when distributed bi-daily to 45:28 min per day, marking a surgery end time of 16:30:28 and more than doubling the 5th case success rate to 2.66%. Finally, given that 4-joint days are only run 4 days a week, the extra time is once again doubled when pooling them for a weekly 5th case. Doing so yields 90:56 extra minutes per 5th case day, for a surgery end time of 17:15:56 and predicted success rate of 26.25%. However, because an end time of 18:00 (135 extra minute) has a predicted success rate of 100%, one week can be skipped to split its extra time among the following two weeks and lead to two days with a predicted 100% 5th case success rate every 3 weeks. Ultimately, this sums up to approximately 35 extra cases per year at no extra cost. A map of potential distributions at a 77% success rate is shown in Figure 9.

**Figure 10:**
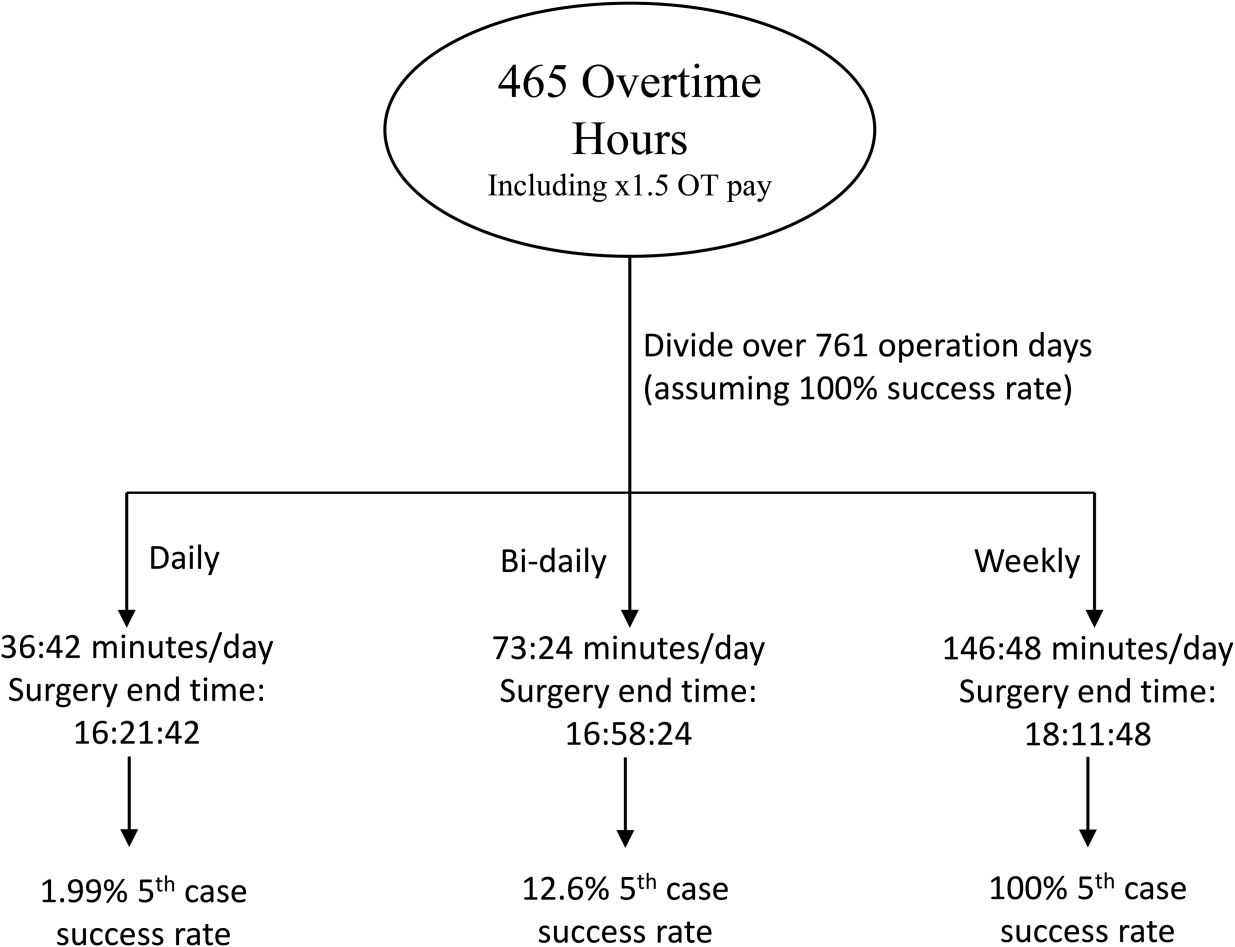
A schematic demonstrating the division of saved overtime-cost hours from performance at a 100% success rate.

Under the situation where a 100% success rate is achieved, all 465 h:22 m:30 s overtime-cost hours would be saved. With a daily split, this amounts to 36 m:42 s per 5th case day, an end time of 16 h:21 m:42 s, and a predicted 5th case success rate of 1.99%. Distributed bi-daily, these values increase to 73 m:21 s, 16:58:24, and 12.6% respectively. When divided weekly, 146:48 extra minutes, adding to an end time of 18:11:48, are given per day, producing a predicted success rate of 100% with a minimum of 10 min to spare. These spared minutes can be pooled to contribute to another 4 cases per year, leading to a total of 56 potential cases per year (assuming the hospital runs all year long). A schematic showing these results is found at Figure 10.

## 10. Discussion

Considering poor 4-joint day success rates, our previous work sought the development of an AI model that provides benchmarks to achieve a certain success rate. Given our institutions' current wait list issues with hip and knee replacement surgeries, 5th cases were predicted to evaluate the potential of their addition using only overtime savings from an increase in success rate. This can lead not only to better staffing efficiency, but higher surgical throughput to help reduce the waiting list as well.

After completing 5th case out of room time predictions, a mean out of room time of 17:24:17 and a median of 17:29:30 were predicted. A negatively skewed distribution was expected due to the same trend with 4th case out of room times. The predictions' standard deviation of 22 min and 10 s also falls within expectation, as it increased relative to previous ground truth cases, as shown in Table 5. Despite the average successful case time of an hour and 59 min, not all predicted 5th cases (85.7%) fall below the 2-h mark (17:45) while all 100% are predicted to end before 18:00, as the latest predicted time is 17:56:30. These findings highlight the presence of nuances in trying to find the optimal balance between time added and prevention of further overtime waste.

When applying the predictions to evaluate how cost-savings can be used to fund 5th cases, two contexts are considered: performance at a 77% success rate, our baseline rate that is deemed achievable and realistic by the clinicians (based on the model's benchmarks), and performance at a 100% success rate, which is the ideal, best-case scenario. In both cases, it seemed preferable to pool the hours at different intervals in order to maximize throughput while minimizing the risk of overtime costs.

At a 77% success rate, the time saved would lead to sub-3% success rates when divided daily or bi-daily. Although this would lead to much higher throughput, it would do so at the cost of many overtime hours, where salary is increased by a factor of 1.5. This case is also true when dividing the hours weekly, as 90 extra minutes leads to a 5th case success rate of only 26.25%. Intuitively, this gives the impression that extra time can be pooled further to guarantee one extra case per month with no extra cost; however, simply adding another 45 min is enough to guarantee success based on the predictions, something that can be done for two weeks by skipping one. In other words, when distributing hours to one day a week, skipping one week leads to a 100% 5th success rate in the following two. Overall, this means that approximately 35 extra cases per year at our institution can be funded solely from the savings accrued by increasing performance to a 77% success rate.

When performing optimally (100%), results were similar, as pooling the saved costs also drastically reduced 5th case overtime costs. Daily and bi-daily distribution of saved costs yielded 5th case success rates of 1.99% and 12.6% respectively, while weekly pooling of saved costs allowed for 100% success rate, with a minimum 10 min to spare. In total, savings can be optimized to project 56 extra cases per year, meaning that 56 cases worth of overtime-cost hours are currently being spent due to inefficient performance at our institution (39.55% success rate).

However, one limitation with our output is the inability to conclusively measure prediction accuracy due to the lack of ground truth 5th case data. Instead, existing data was leveraged to infer the models' accuracy. The first method of doing so, training models to predict 3rd and 4th cases, yielded mean absolute error values of 13 m:40 s and 14 m:13 s minutes respectively. Based on the histograms, most errors are smaller than the means (see Figure 7), with a few large outliers. This is deemed acceptable as these errors can represent variations in case durations that exist in the dataset, making a more representative distribution of predictions. The second method looked instead at how well linear regression fit to the existing data, yielding a mean absolute error value of 4 m:45 s. As with the previous method, the distribution of errors shows a positive skew, indicating that most of the errors are below the mean with a few large outliers. Overall, linear regression fit well to the trends of the dataset. However, as mentioned, the lack of any ground truth 5th case data makes this evaluation inferential, as a more direct evaluation cannot be made.

Another limitation is the uncertainty of how the data might change once the AI model (9) is implemented. Whether use of the model would work by improving the speed of all cases, reducing the number of slow cases, or simply streamline case durations so that they are more consistent is unknown, and could impact the distribution of predictions. Fortunately, it is likely that the use of the model would shift the distribution to the left, potentially making the current evaluation a pessimistic one.

Despite these limitations, we propose a simple, effective, and reproducible method of calculating potential throughput gains with no extra cost as a result of improved performance efficiency. In our case, this improvement relies on the success of a benchmark-establishing AI model developed by members of our team. Furthermore, the gains are only attainable with the modification of staffing procedures so that longer days are had without spending overtime rates; one example for this is to benefit from staff that show up late, and who can stay late, by having them stay longer for the fifth case. This work also opens many avenues of future research: reproduction of this work after implementation of the AI model may produce further refinements to cost-free throughput enhancement depending on how the model affects all cases, and whether it improves successful case durations as well. Research into how staffing can best be modified to account for extra cases could also offer another level of optimization, and a potential area of healthcare reform.

## 11. Conclusion

Due to the COVID-19 pandemic, the Canadian healthcare system was burdened with long hip and knee replacement wait lists and extra costs as a result of cancelled procedures. We aimed to leverage the savings that would be accrued from the use of our AI model to increase surgical throughput with no extra costs. To do this, linear regression models were used to predict 5th case out of room times that served as benchmarks to estimate success rates at different 5th case success times. Success times were determined by distributions of hypothetical overtime-cost savings that would be accrued using the AI model. Previously, our institution operated at a 39.55% success rate. Overall, it was found that increasing to a 77% rate can lead to approx. 35 extra cases per year funded solely by the savings acquired, while operating at a 100% success rate can lead to 56 additional cases per year. Future work can look at the optimization of staffing procedures to account for extra hours with no overtime pay, and the effects of the AI model on all case durations.

## Data Availability

The datasets presented in this article are not readily available because Data has not gone through institutional hurdles to make it open source and available for all. Requests to access the datasets should be directed to FA, falzo100@uottawa.ca.
